# A method for the characterisation of microplastics in sludge

**DOI:** 10.1016/j.mex.2019.11.020

**Published:** 2019-11-19

**Authors:** Pablo Campo, Anita Holmes, Frederic Coulon

**Affiliations:** Cranfield Water Science Institute Cranfield University, Cranfield, Beds, MK43 0AL, UK

**Keywords:** Characterisation of microplastics in wastewater sludge, Sludge, FT-IR, Microplastics, Separation

## Abstract

Microplastics (MP) have become a concern owing to their increasing detection in the environment and potential impact on ecosystems. One of the main MP reservoirs is sludge generated during wastewater treatment. Estimates suggest that, through sludge settling, treatment processes remove between 80 and 90 % of MP present in wastewater. Nevertheless, reliable measurements of actual plastics loads retained by sludge are still lacking for management purposes. Hence, our goal was to validate a quick method for MP quantitation in sludge. Recovery tests were conducted with red low-density polyethylene (LDPE) fragments whose sizes ranged between 5 to 1 mm, 1 to 0.5 mm and 500 to 150 μm. For each size fraction, either 10 or 100 LDPE fragments were spiked into wet sludge (50 mL). Subsequent LDPE analysis involved steps such as freeze-drying, sieving, Fenton purification, visual shorting and FTIR identification. When expressed as number of fragments, quantitative (i.e. percentage values between 80 and 100) were obtained regardless of size fraction or initial spiked number. In terms of total spiked LDPE weight, however, recoveries consistently exceeded 100 % because LDPE fragments retained other materials. Such residues contributed to an overestimation of MP by weight up to 33 % of the 500−150 μm fraction.

• Method was validated by spiking LDPE fragments.
• Recoveries based on MP number showed good precision and accuracy.
• Residues attached to MP resulted in overestimated recoveries by MP weight up to 33 %.

## Graphical abstract

**Figure:**
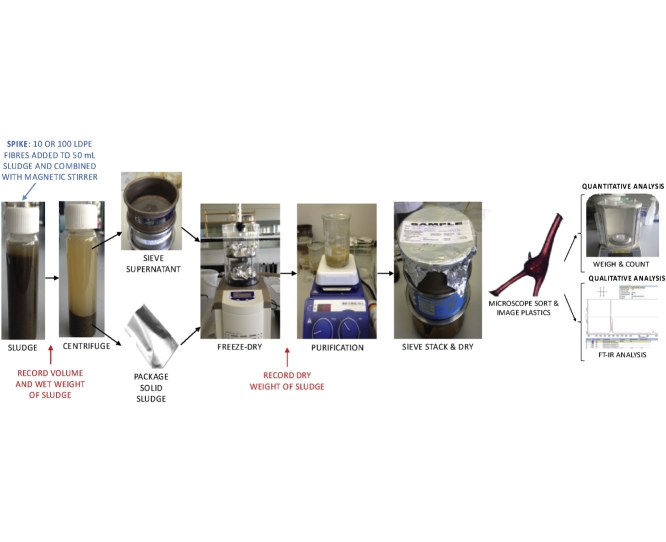

**Specification Table**

**Table:** 

Subject Area:	Environmental Science
More specific subject area:	Microplastics
Method name:	Characterisation of microplastics in wastewater sludge
Name and reference of original method:	NA
Resource availability:	NA

## Method details

### Contamination control

In order to minimise environmental and cross contamination, all steps were conducted in laminar flows hoods. All equipment and containers for storing and measuring the sewage sludge was either glass or metal made. Before use, all wares were rinsed with filtered deionized water. Work surfaces were cleaned with paper towels and a 70 % isopropanol solution. Natural fiber clothes were used at all the times.

### Sample preparation

**Fig. 1:**
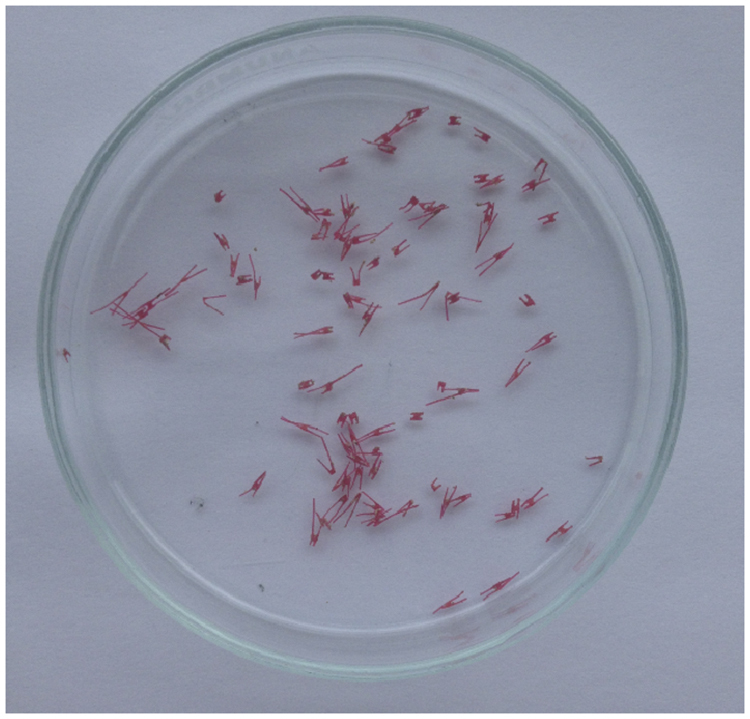
Assorted low-density polyethylene fragments for recovery experiments.

Wet wastewater sludge grab samples (1 L) were collected from the upper section of the sludge holding tank at Cranfield University wastewater treatment plant. Samples were taken from the top layer of the sludge pile with a metal scoop and transferred into 1-L rinsed glass bottles. The sludge samples were mixed in the bottles prior to analysis to prevent the solids settling. To determine the separation efficiency of plastic fragments, wet sludge subsamples (50 mL) were transferred to glass vials and spiked in triplicates with low-density polyethylene (LDPE) fragments, which is the most detected polymer in UK urban river environments [1]. The following size ranges were tested: 5–1 mm, 1–0.5 mm and 500–150 μm. Two spike levels were selected namely, 10 and 100 fragments which corresponded to sample wet weight values of 0.01 and 0.11 %, respectively. The 0.01 % spike replicated a realistic quantity of microplastics in sludge, whereas the 0.11 % spike was applied for a refined indication of the removal efficiency. Spiked subsamples were stirred for 15 min covered with aluminum foil to prevent airborne contamination from clothing and external plastics. All spiked subsamples were run in triplicate and the plastic fragments were counted twice to reduce error. Fig. 1 shows some of the LDPE fragments used in this study.

### Water-sludge separation

For matrix separation subsamples were centrifuged at 700×*g* for 10 min. Supernatants were passed through stack comprising 1 mm, 500 μm and 53 μm sieves, while solid pellets were removed from the vial, packaged in aluminum foil for freeze-drying. Both materials retained in the sieve stack and packaged solids were freeze-dried for 24 h to remove moisture content.

### Purification

Purification of LDPE fragments separated into the supernatant and sludge pellets was done in 250-mL glass beakers by directly adding 20 mL of Fenton’s reagent (0.05 M) and 20 mL of H_2_O_2_ (30 %) to each freeze-dried fraction [2]. Beakers were then placed on magnetic stirrers and heated to 60 °C and left to react for 15 min. Additional 10-mL aliquots of H_2_O_2_ were added after 30 and 90 min and finally the mixture was left to react for 12 h. Once the purification was completed, LDPE fragments from both fractions were recombined by consecutively sieving the respective reaction mixtures.

### Microplastics identification

**Fig. 2:**
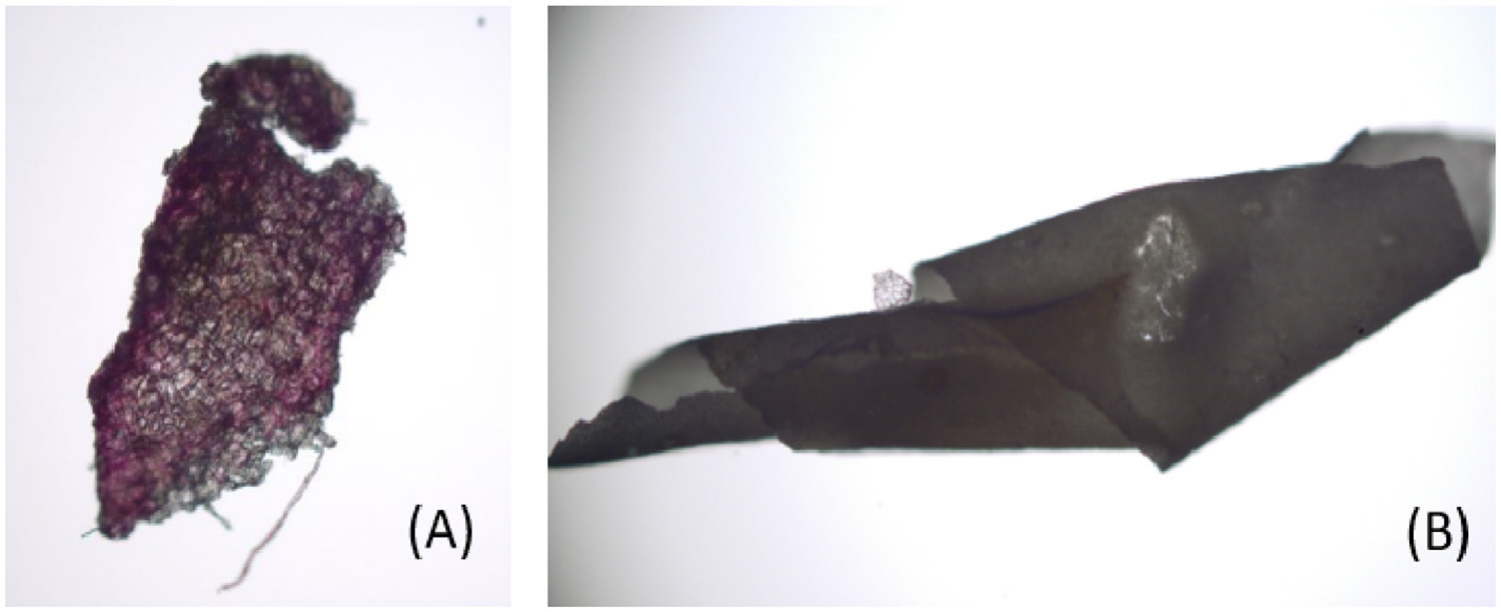
Particle visual shorting after Rose-Bengal stain. Died natural polymer (A) and plastic film (B).

To identify and separate natural polymers (e.g. cotton), Rose-Bengal dye was directly applied to fragments retained on the sieves (Fig. 2) [3]. After a drying stage at room temperature overnight, fragments were isolated under a microscope (Wild Heerbrugg) and tested for structural consistent with tweezers. Subsequently, dyed fragments discharged and microplastics shorted. Further dying of microplastics with Nile red was ruled out since biogenic material (e.g. lipids and chitin) may fluoresce and thus interfere with the identification process [4].

To confirm the polymer was low density polyethylene and establish baseline spectrum data for identification purposes, plastic fragments were analysed by attenuated total reflection FT-IR spectroscopy (Bruker HTS-XT Vertex 70). The instrument was operated at 16 scans with a resolution of 4 cm^−1^. Polymer composition of spiked LDPE fragments was confirmed by matching acquired spectra to the those included in instrument library. With this purpose, three fragments randomly chosen from each subsample were analysed by FTIR.

**Fig. 3:**
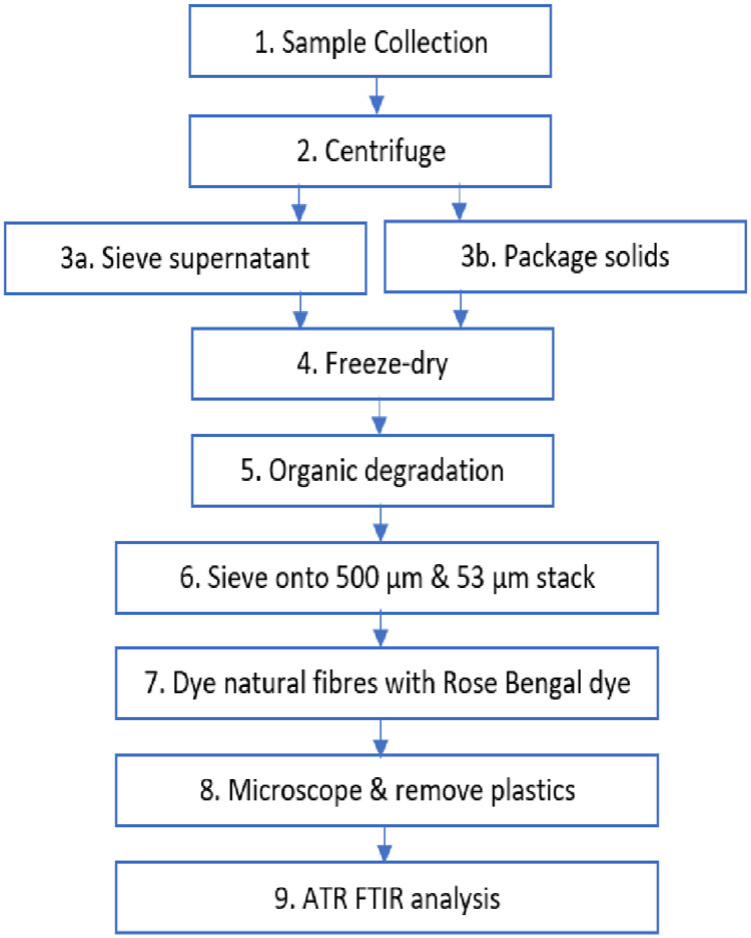
Flow chart of the sequential steps for the analysis of microplastics in wastewater sludge.

Fig. 3 shows the sequential order of the aforementioned analytical steps.

### Method validation

**Fig. 4:**
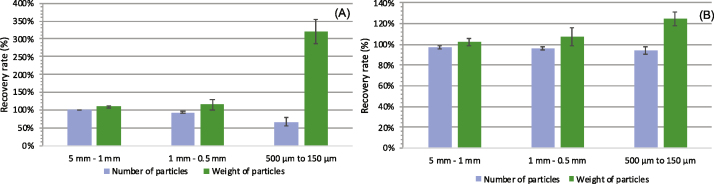
Recovery results of spiked LDPE fragments by number of fragments (blue bars) and total weight of fragments (green bars). Panel A, 10 fragments; Panel B, 100 fragments. (Each value represents mean recovery from triplicate samples and bars indicate standard deviation).

**Fig. 5:**
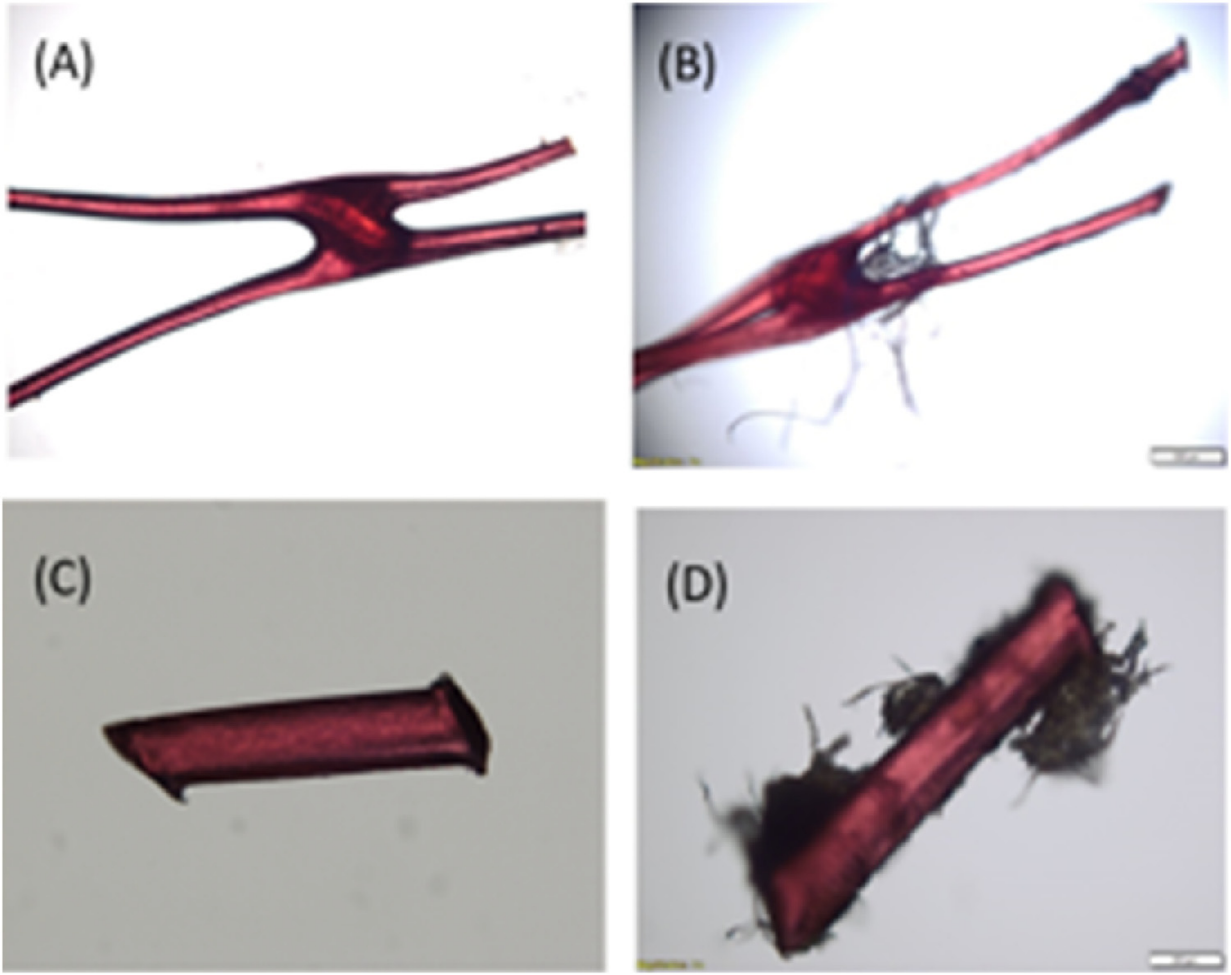
LDPE fragments before (A, C) and after (B, D) spike in wet sludge and further analysis.

As showed in Fig. 4, recoveries based on number of LDPE fragments (blue bars) showed good accuracy regardless of particle number or size fraction. For the 10-particle spike (Fig. 4, panel A), the respective average particle counts for size fractions 5 to 1 and 1 to 0.5 mm were 10 and 9, whereas 7 fragments were recovered for the smallest fraction (500–150 μm). A higher LDPE particle number (i.e. 100-particle spike) translated into consistent results closer to the target value since particle counts consistently attained values of 90 across the three particle sizes (Fig. 4, panel B). When expressed as total LDPE weight (Fig. 4, green bars), recoveries over 100 % were found for both 10- and 100-particle spikes. Materials attached to LDPE fragments contributed to the observed weight gain (see Fig. 5). Residues have a more significant impact on tests conducted with 10 particles and smaller sizes. For instance, a 330 % overestimate was found in the 500 to 150 μm range (Fig. 4, panel A).

Our method yielded comparable recovery values than those obtained after more extensive sample preparation (see Table 1). For instance, Li et al. (2018) used elutriation for removal of the plastics from the sludge [4], this step was not used in our methodology owing to the protentional interference of organic matter that could attach to MPs thus preventing their separation for analysis.

**Table 1:** Microplastics recovery from current results and reported in the literature.

Reference	Matrix	Particle size	Spike quantity (MPs g^−1^ ww)	Recovery
**This study**	Sludge	> 1 mm	2.0	97 ± 1 %
	Sludge	1 mm–500 μm	2.0	96 ± 1 %
	Sludge	500–150 μm	2.0	94 ± 4 %
**Li et al., 2018** [3]	Sludge	550 μm	2.5	86 ± 4 %
	Sludge	75 μm	2.5	67 ± 4 %

The authors whose names are listed immediately below certify that they have NO affiliations with or involvement in any organization or entity with any financial interest (such as honoraria; educational grants; participation in speakers’ bureaus; membership, employment, consultancies, stock ownership, or other equity interest; and expert testimony or patent-licensing arrangements), or non-financial interest (such as personal or professional relationships, affiliations, knowledge or beliefs) in the subject matter or materials discussed in this manuscript.

## Declaration of Competing Interest

The authors whose names are listed immediately below certify that they have NO affiliations with or involvement in any organization or entity with any financial interest (such as honoraria; educational grants; participation in speakers’ bureaus; membership, employment, consultancies, stock ownership, or other equity interest; and expert testimony or patent-licensing arrangements), or non-financial interest (such as personal or professional relationships, affiliations, knowledge or beliefs) in the subject matter or materials discussed in this manuscript.
